# Understanding How and Why Alcohol Interventions Prevent and Reduce Problematic Alcohol Consumption among Older Adults: A Systematic Review

**DOI:** 10.3390/ijerph19063188

**Published:** 2022-03-08

**Authors:** Jogé Boumans, Dike van de Mheen, Rik Crutzen, Hans Dupont, Rob Bovens, Andrea Rozema

**Affiliations:** 1 Tranzo Scientific Center for Care and Wellbeing, School of Social and Behavioral Sciences, Tilburg University, 5000 LE Tilburg, The Netherlands; h.vdmheen@tilburguniversity.edu (D.v.d.M.); r.h.l.m.bovens@tilburguniversity.edu (R.B.); a.d.rozema@tilburguniversity.edu (A.R.); 2 Department of Health Promotion, Care and Public Health Research Institute (CAPHRI), Maastricht University, 6200 MD Maastricht, The Netherlands; rik.crutzen@maastrichtuniversity.nl (R.C.); h.dupont@mondriaan.eu (H.D.)

**Keywords:** alcohol, older adults, interventions, effective elements, realist evaluation

## Abstract

Problematic alcohol use has been increasing in older adults (55+) in recent decades. Many of the effective interventions that are available to prevent or reduce the negative effects of alcohol consumption are aimed at adults in general. It is unclear whether these interventions also work for older adults. The objective of this review was to understand how (i.e., which elements), in which context, and why (which mechanisms) interventions are successful in preventing or reducing (problematic) alcohol consumption among older adults. A systematic review of articles published between 2000 and 2022 was performed using PubMed, PsycINFO, Web of Science and CHINAHL. Realist evaluation was used to analyze the data. We found 61 studies on interventions aimed at preventing or reducing problematic alcohol use. Most of the interventions were not specifically designed for older adults but also included older adults. The findings of the current study highlight three major effective elements of interventions: (1) providing information on the consequences of alcohol consumption; (2) being in contact with others and communicating with them about (alcohol) problems; and (3) personalized feedback about drinking behavior. Two of these elements were also used in the interventions especially designed for older adults. Being in contact with others and communicating with them about (alcohol) problems is an important element to pay attention to for developers of alcohol interventions for older adults because loneliness is a problem for this age group and there is a relationship between the use of alcohol and loneliness.

## 1. Introduction

The World Health Organization has identified alcohol-related harm among older adults as an increasing concern [1]. Researchers in biology often define old age as starting at the chronological age of 55+ because at that age changes in body systems become more evident [2,3].

Over recent decades, alcohol use among older people has increased in several countries, including Spain, the United States, and The Netherlands [4,5,6]. One reason for this is that older adults experience more freedom; that is, they have more time for leisure activities, such as attending social gatherings and participating in clubs, many of which routinely involve alcohol consumption [7,8]. Another reason is that some people use alcohol as a coping strategy to overcome negative changes in physical health and mental health that come with ageing [9,10,11], including increased loneliness and social isolation [12], unemployment and economic downturns [13]. In developing countries, alcohol use has been increasing in line with economic development and global marketing. The increased availability and affordability of alcohol among lower socio-economic groups has also played a role [1,14]. For developing countries, this is in line with increasing economic development, global marketing and the greater availability and affordability of alcohol among lower socio-economic groups [1,14].

The consumption of alcohol, even in small amounts, can cause greater harm among older than younger adults. Alcohol can accelerate and aggravate the onset of conditions associated with aging (e.g., falling hazards [15], cognitive impairment [16] and/or sleep disturbance [17,18,19]). Older adults often receive medication for these conditions. The use of alcohol alongside prescription medications, such as benzodiazepines for insomnia [20], leads to negative interactions, particularly because older adults metabolize and excrete alcohol more slowly [21,22]. The combination of alcohol and prescription medicines could lead to increasing alcohol levels in the blood, reducing the efficacy of medication and exacerbating its side effects [9,23].

The development of effective interventions to prevent or reduce alcohol use in older adults is crucial, not only because of the problems that alcohol consumption causes for older individuals, but also because of the increase in the number of older people. It is expected that, in 2050, one in six people worldwide will be aged 65 years or over [24]. As the population of older adults increases, so will the number of older people who have alcohol-related problems. First, prevention is needed because of the problems that alcohol causes in this group; second, the group of older adults is expanding, resulting in more alcohol-related problems.

Numerous effective interventions have been developed to prevent or reduce alcohol consumption, for example interventions carried out by general practitioners, brief interventions [25,26], psychosocial interventions (e.g., motivational interviewing [27]) and e-health interventions (e.g., web-based interventions [28] and smart phone interventions [29]). However, many of these interventions are aimed at adults in general and not specifically at older adults. It is unclear whether these interventions also work for older adults. Older adults were raised in a different period and may have different norms and values regarding drinking alcohol than young adults [30,31].

Two recent reviews on alcohol consumption among older adults have indicated that interventions to prevent or reduce the negative effects of alcohol consumption in older adults specifically are limited in number. Armstrong-Moore et al. [32] found seven interventions, of which five resulted in alcohol reduction. Kelly et al. [33] identified thirteen studies, of which six reduced alcohol consumption. Most effective interventions include elements of (brief) motivational interventions, (brief) advice or personalized reports on risks and problems. Moreover, it is known only whether the interventions are effective and not which elements of the intervention lead to this outcome or in which context and by which mechanisms. With this information more targeted interventions for alcohol use among older adults could be developed.

### Objectives

To date, no overview is available showing *how* interventions to prevent or reduce alcohol use in older adults work and which are successful for older adults specifically. Therefore, we performed a literature review, following a realist approach, on interventions for (older) adults and extracted the elements of the interventions that were effective. When possible, we also explained why these elements were effective. To understand why an (element of an) intervention leads to the desired outcome, it is also important to understand the context in which the intervention is offered to the target group. Therefore, we also took the context into account. The objective of this review is to understand how (which elements of interventions), in which context and why (by which mechanisms) interventions are successful in preventing or reducing (problematic) alcohol consumption among older adults.

## 2. Materials and Methods

### 2.1. Realist Evaluation Approach

This literature review was informed by the realist evaluation approach. A realist evaluation describes not only the intervention and its outcome (O) but also the context (C) and the underlying mechanism (M) [34]. The context includes elements such as the organizational context, participant features, staffing, and geographical and historical context. Mechanisms are a combination of recourses offered by the (social) program or intervention and human understanding and/or responses to that recourse. Mechanisms are not directly observable and include preferences, reasoning, norms or collective beliefs. Outcomes include changes to people and to their lives, but also include other kinds of alterations (e.g., in organizations, workers or governments) [35]. In the current study, the *interventions* are the programs that help older adults to prevent or reduce their alcohol consumption successfully, the *context* is operationalized as the way in which the intervention is offered to the target group (e.g., digitally, by phone, in-person, individually or in a group setting), the *mechanisms* are the reasons why elements of the interventions work and the *outcome* is the prevention or reduction of alcohol consumption.

### 2.2. Search Strategy

A literature review of peer-reviewed articles published between 2000 and 2020 was performed in April 2020 and updated in February 2022 using PsycINFO, Web of Science (WOS), PubMed and CINAHL (see Table 1). This time span was chosen because the focus on older adults in relation to alcohol issues dates back to the beginning of this century [36]. A combination of five groups of keywords was used to search the databases. These groups of keywords consisted of search terms from all four databases: PsycINFO (thesaurus), Web of Science (no special terms), PubMed (MeSH terms) and CINAHL (heading terms). In addition, synonyms and free text words were used. Four search strings were formed based on the objectives of this review. Due to the scarcity of studies specifically about older adults, we chose to include two groups: a very wide range, which includes older adults (18+), and a specific age range, which only consists of older adults (55+). Table 2 provides an overview of the groups and keywords used. Table 3 provides a summary of the search questions. The review is reported according to PRISMA [37].

### 2.3. Inclusion Criteria

The studies were selected with the following inclusion criteria: (1) studies that focused on interventions for outpatients to prevent or reduce (problematic) alcohol consumption and that mentioned effective elements; (2) the target group of the studies consisted of people aged 18 years or older; (3) peer-reviewed empirical studies published in English after 2000 and available in full text; and (4) studies conducted in Western high-income countries (e.g., Europe, North America, Australia and New Zealand). The exclusion criteria were the following: (1) studies aimed at inpatients; and (2) studies with a very specific target group (i.e., pregnant women, veterans (due to the specific approach of this group, often carried out by the military), ethnic minorities, students, people with an IQ lower than 85 or the forensic target group).

### 2.4. Study Selection

**Figure 1:**
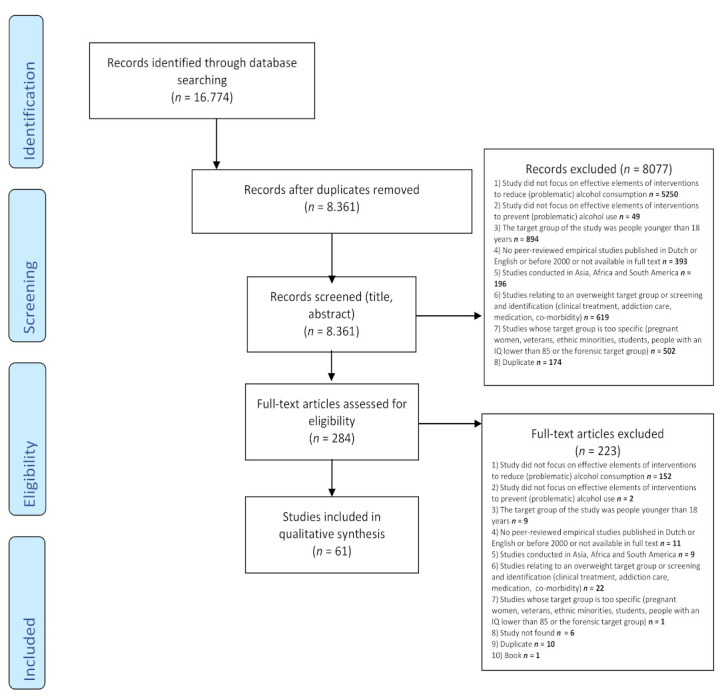
Flowchart.

Based on the inclusion and exclusion criteria, titles, abstracts and full-text articles were screened by the first author (JB). The last author (AR) also screened 20% of all the records (titles, abstracts and full texts). The deviation was less than 10% in all three phases. In the case that JB had doubts about other articles than these 20%, AR was consulted. See Figure 1 for the flowchart of the selection process.

### 2.5. Data Extraction and Analysis

A data extraction form was used, specifying the following information: author(s), title, publication year, study methodology, setting, participants and objective of the study (prevention or reduction), effective elements of the intervention, context, mechanisms and outcome (CMO). Data were extracted by JB and AR independently and discussed thereafter until consensus was reached. A realist evaluation approach was adopted to identify CMO configurations in each study where possible. These configurations described how contextual factors and mechanisms (human responses to elements of interventions or prevention strategies) led to the desired outcomes (prevention or reduction of alcohol consumption). For each study, one or more elements of interventions and/or one or more CMO configurations were drafted. The analyses were performed by JB and AR, focusing on the patterns across elements of interventions and the CMO configurations. The quality of the included studies was assessed using the Mixed Methods Appraisal Tool (MMAT Tool) [38]. The tool includes two screening questions and 19 items for appraising the methodological quality of five categories of studies: qualitative studies, RCTs, non-randomized studies, quantitative descriptive studies and mixed-methods studies. Each study category consists of five items. Each item is rated on a categorical scale (yes, no and cannot tell). The number of items rated “yes” is counted to provide an overall score (0 is low; 5 is high). The appraisal of all the included articles was performed independently by two researchers (JB and AR), and the results were compared; when inconsistencies were apparent, they were discussed until consensus was reached. Studies with a low MMAT score (2 or lower) were only used to support the results found in studies with an MMAT score of 3 or higher.

## 3. Results

### 3.1. Study Selection and Characteristics

We included 61 articles in our review. The characteristics of each included paper are presented in Table 4. A total of 33 studies were quantitative and randomized, 19 studies were quantitative and non-randomized, five studies were qualitative interviews, three studies were quantitative descriptive studies, one study was mixed methods. Of these 61 studies, three described interventions specifically for older adults and were quantitative and randomized studies [39,40,41]. The studies were performed in the following countries: the United States of America (25), the United Kingdom (7), Germany (4), Australia (5), Denmark (4), Canada (4), the Netherlands (4), Spain (2), France (2), Ireland (1), Italy (1), Estonia (1) and New Zealand (1).

The aforementioned three studies [39,40,41] that specifically targeted older adults focused on interventions with personalized feedback and information provision. Interventions for the general populations included therapy sessions, frequently including motivational interviews or motivational enhancement with other educational material. Some interventions offered a stepped care process with personalized feedback on alcohol. The way in which the interventions were delivered differed widely. Personal treatment and the Internet were the most mentioned ways.

The quality assessment results (MMAT score) are shown in Table 4. Overall, the quality of the studies was generally high (4 or 5) or moderate (3). Only three studies were rated low (2) [42,43] or poor (1) [44].

**Table 4:** Characteristics of the studies.

A. Context (C) ^1^: Therapist—In-Person—Individual				How	Why	
Author; Country	Participants; Age Mean (SD)	Method	Intervention or Aim	Intervention Elements (E) ^2^ and Outcome (O) ^4^	Mechanisms (M) ^3^ and Outcome (O) ^4^	Study Quality (MMAT)
**Andréasson et al. (2002); SE** [45]	N = 93; >18+ years Mean age 50.2 years (Not given)	Quantitative randomized controlled trial	*Intervention*: Treatment of alcohol-related problems with cognitive behavioral therapy and motivation enhancement *Control*: One assessment session; one session of feedback/advice, guided by the same motivation enhancement principles; and a 24-page self-help manual	*Intervention*: Four treatment sessions on cognitive behavioral therapy and motivation enhancement (E) → alcohol reduction (O) → reduction in the number of drinking days (O) *Control*: (1) One session of feedback/advice, guided by the same motivation enhancement principles (E) → reduction of alcohol (O)	Not studied	3
**Baumann et al. (2015); DK** [46]	N = 9415; 30–60 years Intervention: mean age 46.1 (7.9) Control: mean age 45.7 (9.8)	Quantitative randomized controlled trial	*Intervention 1A*: Screening, risk assessment and individual lifestyle counselling; participants at high risk of ischemic heart disease were also offered group-based counselling *Intervention 1B:* High-risk people in the intervention group were offered group-based counselling on smoking cessation or on diet and physical activity *Control*: No intervention	*Intervention 1A*: Sessions were conducted by a nurse, dietitian or doctor (E) trained in motivational interviewing (E) → greater reductions in binge drinking during the 5 years of intervention (O) *Intervention 1B*: Additionally, high-risk people in the intervention group were offered group-based counselling on smoking cessation or on diet and physical activity (E) → reported greater reductions in binge drinking during the 5 years of intervention (O)	Not studied	4
**Connors et al. (2016); USA** [47]	N = 63; 18 and 65 years Mean age 48.27 (10.64)	Quantitative non-randomized	*Aim:* Examined therapeutic alliance. Participants seeking treatment for an alcohol use disorder received 12 weeks of cognitive behavioral therapy (CBT) for alcohol dependence and completed weekly assessments of the alliance	Not studied	(1) Higher therapeutic alliance scores (M) (2) achieved though therapist and patient collaboration in the identification of additional sessions as judged best to meet the patient’s clinical needs (M) → fewer drinking days (O) in the period until the next treatment session → fewer heavy drinking days in the period until the next treatment session (O)	5
**Csillik et al. (2022); FR** [48]	N = 45 >18+ years Mean age: 44.6, (11.6)	Randomized controlled trial	*Intervention:* The efficacy of three MI intervention plans using a randomized matched pre-test/post-test design spanning a 10-week period	*Intervention:* Five individual face-to-face motivational interview (E) sessions conducted over a ten-week period → reduction of alcohol consumption (O)	Not studied	3
**Ilgen et al. (2006); USA** [49]	N = 785; Age not specified Mean age not specified	Quantitative randomized	*Aim*: Investigated whether a positive therapeutic relation is particularly beneficial for patients entering alcohol use disorder treatment with low motivation *Intervention*: Project MATCH. Patients were randomly assigned to twelve-step facilitation, cognitive behavioral coping skills or motivational enhancement therapy	Not studied	(1) High-quality therapeutic relationship (M) was more strongly associated with → reductions in alcohol use (O) among patients with (2) low motivation (M) than among those with high motivation	5
**Karno et al. (2002); USA** [50]	N = 47: Age not specified Mean age 38.8	Quantitative non-randomized	*Aim*: Examined the effects of interactions between patient attributes and therapist interventions on alcoholism treatment outcome. The partners of these patients participated in treatment but were not a focus of this study *Intervention:* Psychotherapy session from either cognitive behavioral or family systems therapy	*Intervention*: cognitive behavioral therapy (E) → had significantly better drinking outcomes (O) than family systems therapy (E)	(1) Use of interventions early in treatment that emphasized emotional experiences (M) → less alcohol consumption (O) (2) The relationships between emotional distress and therapist focus on affect (M) and patient reaction and therapist directness (M) → were important predictors of alcohol use (O) during the maintenance phase of treatment	4
**Kavanagh and Connolly (2009); AUS** [51]	N = 204; Age 19–80 years Mean age 47.8 (10.8)	Quantitative randomized controlled trial	*Intervention*: General practitioners (GPs) received a letter providing a summary of baseline assessments plus standard guidelines on management of alcohol disorders in general practice. They were informed of their patients’ progress. Participants received information about alcohol’s effect, a self-help booklet and self-monitoring forms *Control group:* Received information about alcohol’s effects, a self-help booklet and self-monitoring forms. Posted self-monitoring forms each fortnight and received letters that summarized progress, encouraging continued self-monitoring and self-monitoring forms	*Intervention*: GPs receiving information about the alcohol behavior of patients (E) and at monthly intervals over the following 6 months, an update about their patients’ progress (E) → drank on fewer days (O)	Not studied	5
**Khan et al. (2013); UK** [52]	N = 141; >18+ years Mean age not specified	Quantitative non-randomized	*Aim*: Possible benefits of offering a brief alcohol intervention within community pharmacies *Intervention*: Hazardous drinkers received a full brief intervention from the pharmacist based on the Feedback, Listen, Advice, Goals and Strategies (FLAGS) technique	*Intervention*: (1) Full brief intervention given by the pharmacist (E) based on the Feedback, Listen, Advice, Goals and Strategies technique (E) and (2) an alcohol unit wheel calculator (E), (3) a “Units and You” booklet (E) and a leaflet with contact details of local and national specialist alcohol services (E) → reduction in the number of drinking days reported by hazardous drinkers (O) → a highly significant reduction in the number of alcohol units consumed by hazardous drinkers (O)	Not studied	4
*Kiluk et al. (2016); USA [53]	N = 68; >18+ Mean age 42.7 (1.9)	Quantitative randomized controlled trial	*Intervention 1*: Treatment as usual plus on-site access to computerized cognitive behavioral therapy targeting alcohol use *Intervention 2*: Computerized cognitive behavioral therapy plus brief weekly clinical monitoring *Intervention 3*: On-site access to computerized cognitive behavioral therapy targeting alcohol use *Control*: Treatment as usual	*Intervention 1*: Weekly group or individual motivational psychotherapy delivered by masters-level counsellors at the outpatient facility (E) → lower alcohol consumption (O) *Intervention 2*: Computerized cognitive behavioral therapy plus brief weekly clinical monitoring (E) → reduction of alcohol consumption (O) *Control*: (1) Weekly group or individual motivational psychotherapy (E) → reduction alcohol use (O)	Not studied	4
**Kingree and Thompson (2011); USA** [54]	N = 268; >18+ years Mean age not specified	Quantitative non-randomized	*Intervention 1*: Assessed participation in meetings *Intervention 2*: Having a sponsor	*Intervention 1*: Not effective *Intervention 2*: Having a sponsor (E) → subsequent abstinence from alcohol (O)	Not studied	4
**Mowbray (2013); USA** [55]	N = 271; >18+ years Mean age 44.6	Quantitative non-randomized	*Aim*: Could setting drinking goals be a mechanism of change *Intervention 1*: Classic abstinence-based treatment models *Intervention 2*: A drinking programme that helped individuals to reduce, but not to stop, their drinking	*Interventions* (1) Individuals with abstinence as a drinking goal (E) → significantly increased abstinent days (O) → significantly fewer heavy drinking days (O)	Not studied	4
**Nielsen and Nielsen (2018); DK** [56]	N = 276; Intervention: mean age 42.6 Control: mean age 40.3	Quantitative non-randomized	*Intervention*: (1) All patients in the intervention received a single motivational session after assessment and before treatment assignment; (2) patients were allocated to one of the four treatments by the actuarial matching system described above, without discretion for clinical judgement *Control*: Treatment based on clinician judgement	*Intervention*: A single motivational session after assessment and before treatment assignment (E) → significantly more likely to complete treatment and show a greater reduction in drinking (O)	Not studied	3
**Orford et al. (2006); UK** [57]	N = 211; Age not specified Mean age 42 years	Qualitative interviews	*Aim*: To develop a model of change during and following professional treatment (social behavior and network therapy and motivational enhancement therapy) for drinking problems, grounded in clients’ accounts Intervention 1: Three sessions of motivational enhancement therapy over 12 weeks Intervention 2: Eight sessions of social behavior and network therapy over 12 weeks	*Intervention 1*: Not studied *Intervention 2*: Not studied	(1) Thinking differently (M) (2) Family and friends support (M) (3) Acting differently (M) (4) Treatment delivers new insights (5) Down to me: clients often expressed the view that change was self-directed (M) (6) Seeing the benefits (M) (7) Catalyst (M) is a simple summary of a set of processes that were talked about at greater length by clients during pre-treatment interviews → less alcohol consumption (O)	5
**Orford et al. (2009); UK** [58]	N = 397; Age not specified	Qualitative interviews	*Aim*: Social treatment (social behavior and network therapy and motivational enhancement therapy) to explore the factors to which clients attributed positive changes that might have occurred in their drinking Intervention 1: Three sessions of motivational enhancement therapy over 12 weeks Intervention 2: Eight sessions of social behavior and network therapy over 12 weeks	*Intervention 1*: Not studied *Intervention 2*: Not studied	(1) Involvement of other people (excluding therapists or other professionals) in supporting own behavior change by attending treatment sessions or in any other way (M) (2) Communicating better and more openly (M) (3) Awareness of, and thinking about, the consequences of drinking (M) (4) Feedback of results from assessment (M) (5) Thinking about what is important in life (M) → change in drinking (O)	5
**Richardson et al. (2011) NZ** [59]	N = 125; 17–59 years Mean age 37.6 (10.4)	Quantitative randomized controlled trial	*Intervention 1*: Motivational enhancement therapy: four sessions *Intervention 2*: Non-directive reflective listening: four sessions *Control*: No sessions	*Intervention 1*: No significant effect *Intervention 2*: No significant effect *Control*: No significant effect	Therapeutic alliance (M) was significantly higher for clients who attended all four sessions (E) More therapeutic alliance because of more attendance (M) → more abstinent days (O)	3
**Team UR (2005); UK** [60]	N = 742;> 16+ Mean age 41.6 (10.1)	Quantitative randomized controlled trial	*Intervention 1*: Social behavior and network therapy: three 50-min sessions over eight to 12 weeks to help clients build social networks *Intervention 2*: Motivational enhancement therapy comprised three 50-min sessions over eight to 12 weeks, combining counselling in the motivational style with objective feedback	*Intervention 1*: Network therapy to build social networks (E) → reduction of alcohol (O) *Intervention 2*: (1) Counselling in the motivational (E) style and (2) including “significant others” in only the first session to provide only confirmatory information (E) → reduction of alcohol (O)	Not studied	3
* Walitzer and Dermen (2004); USA [61]	N = 64; Male clients mean age of 42.0 years (11.3); Spouses mean age of 39.3 years (9.6).	Quantitative randomized controlled trial	*Intervention 1*: Treatment for problem drinkers only *Intervention 2*: Couples’ alcohol-focused treatment *Intervention 3*: Couples’ alcohol-focused treatment + behavioral couple therapy	*Intervention 1*: Specific strategies for changing drinking patterns and informational lectures on current alcohol and other health-related topics (E) → less alcohol consumption (O) *Intervention 2*: Not applicable see **C. Context: therapist—in-person—relatives** *Intervention 3*: Not applicable see **C. Context: therapist—in-person—relatives**	Not studied	4
**Wiprovnick et al. (2015); USA** [42]	N = 59; Age not specified Mean age 40.25 (11.79)	Quantitative randomized controlled trial	*Intervention 1*: Goal of moderation and a detailed structural personalized feedback module *Intervention 2*: Relational motivational interviewing without directive elements consisting of the non-directive elements of motivational interviewing, including therapist stance (warmth, genuineness, egalitarianism), emphasis on client responsibility for change and avoidance of MI-inconsistent behaviors, such as advising and confronting	*Intervention 1*: Not studied *Intervention 2*: Not studied	The change in therapeutic bond (E) and empathic resonance (M) from Week 1 to Week 8 was → significant in predicting drinking outcomes (O) → and decreased alcohol use at the end of treatment for participants in both conditions (O)	2
**B. Context: Therapist—Not-in-Person—Individual**						
**Author; Country**	**Participants; Age**	**Method**	**Intervention or Aim**	**Intervention Elements (E) ^2^ and Outcome (O) ^4^**	**Mechanisms (M) ^3^ and Outcome (O) ^4^**	**Study Quality (MMAT)**
**Best et al. (2015); USA** [62]	N = 22; >18+ Mean age 43.1 (12.9)	Quantitative descriptive	*Intervention*: A 24-h, 7-days-a-week, free, anonymous state-wide telephone counselling, information and referral service for people who use alcohol and other drugs	*Intervention*: (1) Over the telephone; (2) practice elements were presented in the manual alongside a spatial representation through a cognitive (node-link) mapping exercise. After the initial session, the participants were posted a copy of the workbook containing node-link maps, drink diaries and information connected to each of the relevant modules (E) → reduction in drinking (O)	Not studied	3
**Bischof et al. (2008); DE** [63]	N = 408; 18–64 years Intervention 1: mean age 36.8 (13.2) Intervention 2: mean age 36.8 (13.5) Control mean age 35.9 (13.7)	Quantitative randomized controlled trial	*Intervention 1*: Stepped-care participants received computerized feedback and a maximum of three brief counselling sessions based on motivational interviewing and behavioral change counselling. All counselling sessions were conducted by telephone *Intervention 2*: Full-care participants received computerized feedback and simultaneously received brief counselling sessions conducted by trained psychologists based on motivational interviewing and containing structured elements of behavioral change counselling. Counselling sessions were conducted by telephone *Control group*: Participants received roughly half of the number of intervention in minutes compared with full-care participants	*Both interventions*: (1) Received computerized feedback (E) and (2) a maximum of three brief counselling sessions based on motivational interviewing and behavioral change counselling. (3) All counselling sessions were conducted by telephone (E) → reduction in drinking (O) *Control group*: No significant effect	Not studied	3
**Blankers et al.,** **(2011); NL** [64]	N = 205; 18–65 years Mean age 42.2 (9.7)	Quantitative randomized controlled trial	*Intervention 1*: No-therapist-involved web-based intervention: fully automated, self-guided treatment programme *Intervention 2*: Therapist-involved web-based intervention: synchronous online therapy including up to seven synchronous text-based chat therapy sessions. Before each chat session, the participant worked on a homework assignment. There was no other kind of contact between participants and therapists	*Intervention 1*: (1) Feedback about alcohol consumption is provided with interactive graphs and table (E) → reducing alcohol (O) *Intervention 2*: (1) Online therapy (E); (2) seven synchronous text-based chat therapy sessions (E). (3) Before each chat session, the participant worked on a homework assignment (E) → reduction of alcohol consumption (O)	Not studied	4
**Brown et al. (2007); USA** [65]	N = 897; 21–59 years Mean age not specified	Quantitative randomized controlled trial	*Intervention*: Motivational telephone calls: an adaptation of motivational interviewing administered over up to six telephone sessions *Control*: Received a four-page pamphlet on healthy lifestyles. One page was devoted to each of four topics: tobacco, diet, exercise and alcohol	*Intervention*: (1) Motivational telephone calls bolstered with summary letters (E) → significantly reduced drinking for male primary-care patients with alcohol abuse or dependence who are not necessarily seeking assistance for their drinking (O) *Control*: Four-page information pamphlet on healthy lifestyles (E) → reduction in alcohol consumption in women (O)	Not studied	4
**Clifford et al. (2007); USA** [66]	N = 235;> 18+ years Mean age 40.01 (10.00)	Quantitative randomized controlled trial	*Intervention 1*: Frequent comprehensive (FC) quarterly in-person follow-up interviews interspersed with monthly telephone interviews for a period of 12 months after participants’ treatment programme intake interview session. The content of the FC interviews covered the following areas: drinking and drug-taking behaviors; alcohol- and other drug-related negative consequences; medical and psychiatric status; psychological, social and cohabitation/marital relationships; and occupational functioning *Intervention 2*: Frequent brief (FB) quarterly in-person follow-up interviews interspersed with monthly telephone interviews for a period of 12 months after respondents’ treatment programme intake interview session. However, before the final 12-month, in-person interview, interviews were limited to addressing alcohol and other drug-taking behaviors *Intervention 3*: Infrequent comprehensive (IC) interviews only at the baseline and 6- and 12-month research assessment interviews. The content of the assessment battery was identical to that of the FC condition *Intervention 4*: Infrequent brief (IB) in-person follow-up interviews (i.e., only two, at 6 and 12 months) and a 6-month interview limited to the assessment of alcohol and other drug-taking behaviors	For all interventions: Follow-up contact after treatment with the study participants, even if brief in nature (E) → less alcohol consumption (O) FC and IC: (1) More follow-up contact after treatment for study participants (2) in-person (E) (3) or by telephone (E) → less alcohol consumption (O)	Not studied	5
**Ettner et al. (2014); USA** [39]	N = 1168; Age above 60 years Mean age 71	Quantitative randomized controlled trial	*Intervention*: Project SHARE (Senior Health and Alcohol Risk Education), which included personalized reports, educational materials, drinking diaries, physician advice during office visits and telephone counselling *Control group*: Care as usual	*Intervention*: (1) Mailed a personalized patient report (E); (2) an educational booklet on alcohol and aging (E); (3) via telephone, a health educator contacted intervention patients three times. During these calls, the health educator answered questions about the written materials and gave feedback → at-risk drinkers reduced (O) → less alcohol consumption (O) → older adults were more likely to have discussed their alcohol use with a physician (O)	Not studied	5
**Postel et al. (2015); NL** [67]	N = 144; >18+ 22–66 years Mean age 45.8	Quantitative non-randomized	*Intervention*: A 3-month web-based alcohol treatment programme using intensive, asynchronous (non-simultaneous) therapeutic support at a 9-month follow-up assessment	*Intervention*: The web-based treatment programme consisted of (1) a structured two-part online treatment programme (E); (2) the participant and the therapist communicated asynchronously via the Internet (intensive asynchronous therapeutic) (E) → reduction in the number of drinks per week (O)	Not studied	4
**C. Context: Therapist—In-Person—Relatives**						
**Author; Country**	**Participants; Age** **Mean (SD)**	**Method**	**Intervention or Aim**	**Intervention Elements (E) ^2^ and Outcome (O) ^4^**	**Mechanisms (M) ^3^ and Outcome (O) ^4^**	**Study Quality (MMAT)**
**Doyle et al. (2003); IE** [68]	N = 67; Age not specified Mean not specified	Quantitative non-randomized	*Intervention*: Community-based 10-week programme involved weekly separate and conjoint group therapy for problem drinkers and their families	(1) Clients and their families attended psychoeducational lectures (E) and (2) films on addiction and recovery (E) → abstinent (O) or drinking moderately (O)	Not studied	4
**McCrady et al. (2002); USA** [69]	N = 68; Age not specified Mean age 39.4 (10.3)	Quantitative non-randomized	*Intervention*: Alcohol behavioral couple therapy (ABCT) model; three primary domains are assumed to be related to alcohol consumption: (a) individual factors related to the drinker’s alcohol consumption, (b) the quality and nature of the spouse’s responses to alcohol-related situations and (c) the nature and quality of the couple’s marital interactions	*Intervention*: Greater spousal use of problem solving and social support to deal with problems and less use of self-blame, wishful thinking and avoidance → less intense drinking during treatment (O)	*Intervention*: (1) The quality of the pre-treatment marital relationship (M) → men’s ability to remain abstinent (O) (2) The degree of the respondents’ marital happiness immediately after treatment (M) → predicted the intensity of their drinking (O)	4
**McCrady et al. (2009); USA** [70]	N = 102; Age not specified Intervention 1: mean age 44.78 (9.14) Intervention 2: mean age 45.31 (9.31)	Quantitative randomized controlled trial	*Intervention 1:* Alcohol behavioral couple therapy (ABCT) manual-guided, 20-session outpatient cognitive behavioral therapies with an explicit goal of abstinence from alcohol; all sessions included both partners	*Intervention 1*: (1) Sessions included both partners in all sessions (E) and (2) included self-monitoring, functional analysis of drinking and coping skills to avoid alcohol and deal with other life problems (E) resulting in more days abstinent (O) → fewer days heavy drinking (O)	*Intervention 1*: Interventions to teach the partner to support abstinence and to decrease attention to drinking and interventions to improve the couple’s relationship, including reciprocity enhancement, communication and problem solving (M) → more days abstinent (O) → fewer days heavy drinking (O)	4
**Rentscher et al. (2017); USA** [71]	N = 33; Age not specified Mean age 39.2 (10.2)	Quantitative non-randomized	*Aim*: Investigating pronoun use prior to and during two couple-focused interventions for problematic alcohol use: cognitive behavioral therapy and family systems therapy	*Intervention*: Not studied	Spouse we-talk (M) → associated with successful treatment outcomes (O)	3
**Schumm et al. (2014); USA** [72]	N = 105; 18–65 years Mean age women 44.42 (8.08) men 47.68 (8.40)	Quantitative randomized controlled trial	*Intervention 1*: BCT (behavioral couple therapy) sessions attended together by the woman and her partner *Control*: IBT (individually-based therapy) for women	*Intervention*: (1) 13 BCT sessions attended together by the woman and her partner (E) (2) to build support for abstinence and improve relationship functioning (E); (3) completion of a daily “trust discussion” in which the patient states an intent to stay abstinent that day and the spouse expresses support for the patient’s efforts (E) → more abstinent days during treatment and during the 12-month follow-up (O)	*Intervention*: (1) Teaching partners to decrease behaviors that may trigger or enable substance use (M); and (2) helping the couple to decrease the patient’s exposure to alcohol and drugs by removing alcohol from the home and avoiding or managing alcohol-related family and social gatherings (M) → more abstinent days during treatment and during the 12-month follow-up (O)	3
**Vedel et al. (2008); NL** [73]	N = 64; Age not specified Mean age 45.5 (11.34)	Quantitative randomized controlled trial	*Intervention 1*: Behavioral couples therapy *Intervention 2*: Cognitive behavioral therapy	*Intervention 1*: (1) Individual couple sessions (E); (2) 10 sessions (E); (3) 90 min (E) → reduction in drinking (O) *Intervention 2*: Cognitive behavioral therapy (1) emphasizes overcoming skill deficits and aims to increase the person’s ability to detect and cope with high-risk situations that commonly precipitate relapse (E) → reduction of drinking (O)	Not studied	4
* Walitzer and Dermen (2004); USA [61]	N = 64; Age not specified Male clients mean age 42.0 (11.3); Spouses mean age of 39.3 (9.6).	Quantitative randomized controlled trial	*Intervention 1*: Treatment for problem drinkers only (PDO) *Intervention 2*: Couples’ alcohol-focused treatment; subjects were presented with specific strategies for changing drinking patterns; informational lectures on current alcohol and other health-related topics *Intervention 3*: Couples’ alcohol-focused treatment + behavioralcouple therapy; subjects were presented with specific strategies for changing drinking patterns	*Intervention 1*: Not applicable see **A.Context (C) ^1^: therapist—in-person—individual** *Interventions 2 +3* (not specified): A significant increase was obtained in the frequency of drinking days (O)	*Intervention 2*: Treatment material for the alcohol-focused spouse involvement component, presented in conjunction with the client’s drinking reduction strategies, consisted of specific strategies designed to increase spouse behaviors supportive of drinking reduction and to support the problem drinker’s independence and autonomy (M) → reduction in alcohol use (absent and light drinking days) (O) → reduction in heavy drinking days (O) *Intervention 3*: BCT consisted of a series of treatment components designed to equip each couple with a variety of skills and techniques (a) to increase cohesion and the positive aspects of their marriage and (b) to enhance communication and conflict resolution skills (M) → reduction in alcohol use (absent and light drinking days) (O) → reduction in heavy drinking days (O)	4
**D. Context: Therapist—In-Person—Group Component**						
**Bamford et al. (2003); UK** [74]	N = 124; 21–64 years Mean age 41	Quantitative non-randomized	*Intervention*: Short 6-week intervention that focused on psycho-educational materials on physical and mental complications	*Intervention*: (1) Focused on psycho-educational materials on physical and mental complications (E); (2) coping with family problems and mistrust (E); (3) visitors’ groups, in which patients who had made positive changes to their drinking described their experiences (E) and positive influences (E) and (4) spent less time on problem solving and managing low mood and anxiety → reduced drinking behavior (O)	Not studied	4
**Brown (2007); CA** [75]	N = 76; Age not specified Intervention: mean age 41.0 (9.9) Control: mean age 33.2 (8.7)	Quantitative non-randomized	*Intervention*: Brief, four-session group-adapted motivational interviewing (GAMI) *Control*: Standard care (SC)	*Intervention*: (1) Standardized, four-session group treatment (E) (2) in a brief, four-session GAMI intervention (E) → alcohol reduction (O)	*Intervention*: (1) Targeting rapid internally motivated change (M); (2) all sessions were conducted using the specific communication style and strategies associated with motivational interviewing (M) → alcohol reduction (O)	5
**Gómez- Recasens et al. (2018) ES** [76]	N = 1103; >18+ years Mean age 42.48 (10.44)>	Quantitative non-randomized	*Intervention*: To promote health and prevent alcohol and drug consumption in the workplace, emphasizing (1) health promotion and health monitoring, which included (a) alcohol and drug awareness and (b) the evaluation and monitoring of alcohol and drug consumption through a semi-structured interview designed to assess risky consumption; urine tests aimed at detecting alcohol, cannabis and cocaine use; an Alcotest based on expired air to test for the recent consumption of alcohol and a saliva exam to test for the recent consumption of six drugs; and (2) secondary prevention if risky consumption was identified	(1) Awareness (E), (2) information (E), (3) training (E), (4) participation in a workshop outside work (E), (5) evaluation and health surveillance (E), (6) medical examination (E), (7) brief intervention (E), (8) personalized advice (E), (9) personalized follow-up (E), referral to the centre for the attention and monitoring of drug addictions (E) → reduced risky alcohol consumption (O)	Not studied	4
**Hagger et al. (2011); UK** [77]	N = 281; 18–65 years Mean age 35.65, (12.44)	Quantitative randomized controlled trial	*Intervention*: Mental simulation manipulation in pen-and-paper form after receiving information about reducing alcohol consumption and questionnaire measures *Control*: Received identical measures and information about alcohol consumption	*Intervention*: (1) Mental simulation exercise (E) (2) about alcohol intake (E) and (3) health benefits of keeping alcohol intake within guidelines limits (E) → consuming fewer unit of alcohol during the 4-week follow-up period (O) *Control*: Effect not studied	Not studied	4
**Reynolds and Bennett (2015); USA** [78]	N = 1510; >18+ years Mean age not specified	Quantitative randomized controlled trial	*Intervention 1*: The Team Awareness Program: (1) peer referral and (2) team building: 4-h on-the-job classroom training sessions that encouraged healthy lifestyles and the seeking of professional help *Intervention 2*: The Choices in Health Promotion Program delivered various health topics based on a needs assessment: 4-h on-the-job classroom training sessions that encouraged healthy lifestyles and the seeking of professional help	*Intervention 1*: (1) Relevance (E); (2) team ownership of policy (E); (3) understanding tolerance (E); (4) communication (E); (5) support and encourage help (E) → reduced monthly alcohol intake (O)*Intervention 2*: (1) 4-hour programme developed based on needs assessment (E), (2) goal setting (E) and (3) choice components (E) → reduced monthly alcohol intake (O)	Not studied	3
**Toft et al. (2009); DK** [79]	N = 9.415; 30, 35, 40, 45, 50, 55 and 60 years Majority of individuals at the age of 40 to 50 years	Quantitative randomized controlled trial	*Intervention 1* (*Low risk*): Each participant had a lifestyle consultation focusing on smoking, physical activity, diet and alcohol *Intervention 2* (*High risk*): Each participant had a lifestyle consultation focusing on smoking, physical activity, diet and alcohol. The individually counselled high-risk individuals were offered group counselling on diet and exercise or smoking	*Intervention 1*: (1) Each participant had a lifestyle consultation focusing on smoking, physical activity, diet and alcohol (E) → men decreased their alcohol intake (O) → less binge drinking in both men and woman (O) *Intervention 2*: (1) Each participant had a lifestyle consultation focusing on smoking, physical activity, diet and alcohol (E); (2) the individual counselling high-risk individuals were offered group counselling on diet and exercise or smoking; (3) the relatives of the participants were offered the chance to participate in one of the meetings (E) → men decreased their alcohol intake (O) → less binge drinking in both men and women (O)	Not studied	4
**E. Context: No Therapist—Not In-Person—Individual**						
**Author; Country**	**Participants; Age** **Mean (SD)**	**Method**	**Intervention or Aim**	**Intervention Elements (E) ^2^ and Outcome (O) ^4^**	**Mechanisms (M) ^3^ and Outcome (O) ^4^**	**Study Quality (MMAT)**
**Augsburger et al. (2021); EE** [80]	N = 589 >18+ years Mean age: 37.86 (11.16)	Randomized controlled trial	*Intervention:* To estimate the efficacy of an on-line self-help intervention to reduce problem drinking at thepopulation level	*Intervention:* 10 modules based on principles of cognitive–behavioral therapy and motivational interviewing (E). Access to a website with a self-test including personalized normative feedback (E) and information for standard alcohol treatment. Control: access to a help-page received PNF on a self-test for alcohol consumption (E) and furtherinformation together with contact details for treatment options → reduction of alcohol consumption (O)	Not studied	3
**Baumann et al. (2017); DE** [81]	N = 1282; 18–64 years Mean age 30.1 (11.1)	Quantitative randomized controlled trial	*Intervention 1*: Brief intervention tailored to the motivational stage (ST) *Intervention 2*: Brief non-stage tailored intervention (NST) *Control*: Assessment only (AO)	*Intervention 1*: (1) Individualized computer-generated feedback letters in comparison to other persons at the same stage of change and feedback on intrapersonal changes by comparing the participant’s current and previous data (E); (2) the letters referred to particular pages in the accompanying stage-matched manual for further information) → only persons with daily low use benefitted from intervention (O) → more change of being abstinent after 15 months (O) *Intervention 2*: Individualized computer-generated feedback letters in comparison to other persons at the same stage of change and feedback on intrapersonal changes by comparing the participant’s current and previous data (E) → only persons with daily low use benefitted from intervention (O) → more chance of being abstinent after 15 months (O) *Control*: No effect given	Not studied	4
**Bagnardi et al. (2011); I** [82]	N = 6026; >15+ Mean age not specified	Quantitative non-randomized	*Intervention* (*coordinated community-based intervention*): Informing residents about and committing them to the project, brochures, alcohol-free parties, public events promoting a healthy lifestyle, news about the project in local newspapers educating at schools, religious and sporting facilities, meetings with parents/teachers, driving schools, physicians, police forces and volunteers and meetings and alcohol-free events at centres for older adults	*Intervention*: (1) Informing residents about and committing them to the project (E), brochures (E), alcohol-free parties (E), public events promoting a healthy lifestyle (E), news about the project in local newspapers (E); (2) educating at schools, religious and sporting facilities (E), meetings with parents/teachers, driving schools, physicians, police forces and volunteers (E) and meetings and alcohol-free events at centres for older adults (E) → reduced alcohol consumption (O)	Not studied	3
**Blankers et al.,** **(2011); NL** [64]	N = 205; 18–65 years Mean age 42.2 (9.7).	Quantitative randomized controlled trial	*Intervention 1*: No-therapist-involved web-based intervention: fully automated, self-guided treatment programme *Intervention 2*: Therapist-involved web-based intervention: synchronous online therapy including up to seven synchronous text-based chat therapy sessions. Before each chat session, the participant worked on a homework assignment. There was no other kind of contact between participants and therapists	*Intervention 1*: Feedback about alcohol consumption was provided with interactive graphs and table (E) → reducing alcohol conspumtion (O) *Intervention 2*: (1) Online therapy (E), (2) seven synchronous text-based chat therapy sessions (E), (3) before each chat session, the participant worked on a homework assignment (E) → reduction of alcohol consumption (O)	Not studied	4
**Connors et al. (2017); USA** [83]	N = 111; Age not specified Woman: mean age 46.99 (11.79) Men: mean age 15.54 (2.70)	Quantitative randomized controlled trial	*Intervention 1*: Bibliotherapy (a self-directed manual) alone *Intervention 2*: Bibliotherapy with one telephone-administered motivational interview *Intervention 3*: Bibliotherapy with one telephone-administered motivational interview and six biweekly telephone (50 min) therapy sessions	*Intervention 1*: (1) 122-page self-directed manual (E) (2) focused on self-awareness of drinking behavior, identifying danger signals regarding problem drinking situations, developing strategies for reducing alcohol intake and reducing risks associated with drinking that does occur → fewer heavy drinking days (O) → increased abstinent and light drinking days (O) *Intervention 2*: Bibliotherapy with one telephone-administered motivational interview of 60 min (E) → increased abstinent and light drinking days (O) *Intervention 3*: Bibliotherapy (E) with one telephone-administered motivational interview (E) and six biweekly telephone (50 min) therapy sessions (E) → increased abstinent and light drinking days (O)	Not studied	4
**Cunningham et al. (2001); CA** [84]	N = 449; Age not specified Intervention: mean age 41.0 (10.8) Control group: 38.8 (10.5)	Quantitative non-randomized	*Intervention*: Brief self-help booklet provided at assessment for alcohol treatment	*Intervention*: (1) Self-help booklet (E); (2) perspective of encouraging the individuals to consider the costs of their drinking, to motivate them to want to change and to take the next step towards change (E) → drinking on fewer days (O) → and drinking less on each occasion (O)	Not studied	4
**Cunningham et al. (2009); CA** [85]	N = 185; Age not specified Mean age 40.1 (13.4)	Quantitative randomized controlled trial	*Intervention* (*internet personalized alcohol feedback*): After completing a brief online assessment, participants received a ‘Personalized Drinking Profile’ *Control*: Sent a list of the informational components that could be included in a computerized summary for drinkers	Intervention: (1) Participants received a ‘Personalized Drinking Profile’ (E); the core element was normative feedback pie charts that compare the participant’s drinking with that of others of the same age, sex and country of origin (E) → less alcohol consumption (O)	Not studied	4
**Cunningham et al. (2014); CA** [86]	N = 741; >19+ years Mean age 29.8 (9.7)	Quantitative randomized controlled trial	*Intervention 1*: The normative feedback component of the Check Your Drinking Screener (a personalized feedback intervention) *Intervention 2*: Personalized feedback information of the Check Your Drinking Screener *Intervention 3:* The full Check Your Drinking Screener intervention, both the normative feedback and other personalized feedback components. *Control*: No intervention	*Intervention 1*: No significant effect *Intervention 2*: (1) Personalized feedback information (E) → reduction in the number of drinks in a typical week (O) *Intervention 3*: (1) Both the normative feedback (E) and other personalized feedback components (E) → reduction in the number of drinks in a typical week (O)	Not studied	4
**Dulin et al. (2014); USA** [87]	N = 28; 18–45 years Mean age 33.6 years, (6.5)	Quantitative descriptive	*Intervention*: Smartphone-based intervention: stepwise approach to providing the information and interventions to the client; enhancement of motivation for change by providing assessment feedback and immediate coping strategies	*Intervention*: (1) Enhancement of motivation for change by providing assessment feedback (E); fewer heavy drinking days (O) → fewer drinks per day (O)	*Intervention*: (1) Enhanced awareness, i.e., “it helped me to keep track” and “the reports made me realize how much I was drinking and what were my triggers” → fewer heavy drinking days (O) → fewer drinks per day (O)	4
**Fink et al. (2005); USA** [40]	N = 711; >65+ Mean age 75.6	Quantitative randomized controlled trial	*Intervention 1*: Combined report intervention: participants and their GPs received a personalized report of their drinking risks and education *Intervention 2*: Patient report intervention: only participants received a personalized report of their drinking, risk and education *Control:* Minimal assessments	*Interventions 1 and 2*: (1) Personalized reports of their drinking classification (E) and (2) educational information to patients (E) → reduction alcohol (O)	Not studied	5
**Freyer-Adam et al. (2014); DE** [43]	N = 1243; 18–64 years Control: mean age 30.1 (10.9) Intervention 1: mean age 29.5 (10.7) Intervention 2: mean age 30.6 (11.7)	Quantitative randomized controlled trial	*Intervention 1*: The stage tailored intervention: individualized computer-generated feedback letters and self-help manuals. Each text module was dependent on the current stage of change *Intervention 2*: The non-stage tailored intervention: individualized computer-generated feedback letters and self-help manuals	*Intervention 1*: (1) individualized computer-generated feedback letters (E); (2) each text module was dependent on the current stage of change (E); (3) the participant’s responses were compared with normative data of individuals at the same stage (E); (4) self-help manuals (E) → reduction of alcohol consumption in the short term (O) *→* reduction of alcohol consumption in the long term (O) *Intervention 2*: (1) Computer-generated feedback letters (E); (2) feedback was accompanied by information and/or advice; (E) (3) participants were encouraged to complete a when/where/how-to-change plan, introduced by gender-specific examples (E) → reduction in alcohol consumption in the short term (O)	Not studied	2
**Giroux et al. (2014); USA** [88]	N = 28; 22–45 years Mean age 33.6 (6.5)	Qualitative interviews	*Intervention*: Smartphone-based intervention: 10 psychoeducational modules and tools for change, which provided immediate coping strategies and monitoring functions for numerous alcohol-related issues	*Intervention*: Not studied	Smartphone-based intervention (1) raising awareness about drinking (M) → motivated to change their drinking (O) (2) teaching new skills that could be transferred to other areas of their life (M) → motivated to change their drinking (O) → (3) tracking progress related to their goals → motivation to continue engaging in non-drinking behavior (O)	5
**Gonzalez and Dulin (2015); USA** [89]	N = 60; >18+ years Intervention 1: mean age 33.57 (6.54) Intervention 2: mean age 34.30 (6.22)	Quantitative non-randomized	*Intervention 1*: Location-Based Monitoring and Intervention for Alcohol Use Disorder (LBMI-A): participants were provided with a customized LBMI-A-enabled smartphone. The LBMI-A provided seven psychoeducation modules or steps: (1) assessment and feedback, (2) high-risk locations for drinking, (3) selecting and using supportive people for change, (4) cravings and their management, (5) problem-solving skills, (6) communication and drink refusal skills and (7) pleasurable non-drinking activities. Following the completion of a step, an associated tool became available *Intervention 2*: The online Drinker’s Check-Up plus bibliotherapy (DCU+Bib): the DCU is an internet-based, brief motivation intervention that can be completed in less than one hour. It provides a comprehensive assessment of drinking and alcohol-related problems, objective and norm-based feedback, a decisional balance exercise to help resolve ambivalence about change, goal selection, brief development of a change plan and brief interventions to facilitate change	*Intervention 1*: (1) Seven psychoeducation modules (E); weekly feedback reports (E) → less alcohol consumption (O) → fewer heavy drinking days (O) *Intervention 2*: (1) Objective and norm-based feedback (E); (2) links to other online interventions and resources (E); (3) a 16-page booklet (E); (4) an accompanying web page that has additional interactive worksheets and modules for handling urges, drink refusal and recovering from a slip (E) → less alcohol consumption (O) → fewer heavy drinking days (O)	Not studied	3
**Guillemont et al. (2017); FR** [90]	N = 1147; >18+ years Mean age not specified	Quantitative randomized controlled trial	*Intervention*: The Alcoometre self-help web-based intervention delivers personalized normative feedback and some general information about alcohol *Control*: Were informed that their alcohol consumption was hazardous and were given information about hazardous drinking	*Intervention*: (1) Web-based intervention (E) delivers (2) personalized normative feedback (E) and (3) some general information about alcohol (E). (4) Participants can review their motivations and fears regarding reducing their alcohol intake (E), (5) set individual goals (E) and (6) monitor their progress via a consumption diary and other tools (E) → reduction in weekly alcohol intake (O)	Not studied	3
* Kiluk et al. (2016); USA [53]	N = 68; >18+ years Mean age 42.7 (11.9)	Quantitative randomized controlled trial	*Intervention 1*: Treatment as usual plus on-site access to computerized cognitive behavioral therapy targeting alcohol use *Intervention 2*: Computerized cognitive behavioral therapy plus brief weekly clinical monitoring *Intervention 3*: On-site access to computerized cognitive behavioral therapy targeting alcohol use *Control*: Treatment as usual	*Intervention 1*: Not applicable see **C)1: therapist—_in-person—_individual** *Intervention 2*: Not applicable see **C)1: therapist—_in-person—_individual** *Intervention 3*: On-site access to computerized cognitive behavioral therapy targeting alcohol use → reduction of alcohol consumption (O) *Control*: Not applicable	Not studied	4
**Koffanus (2018); USA** [91]	N = 40; >18+ years Intervention: mean age 46.6 (12.5) Control: mean age 45.2 (11.5)	Quantitative randomized controlled trial	*Intervention*: Breathalyser that allows remote, user-verified collection of a breath alcohol sample, text messaging and reloadable debit cards for remote delivery of incentives to evaluate a contingency management treatment for alcohol use disorder that can be delivered with no in-person contact	*Intervention*: (1) 21 consecutive days with three remote breathalyser screens per day (E); (2) participants self-reported their previous day’s alcohol use and current withdrawal symptoms daily in response to a text message and/or phone call (E); (3) participants chose these times each day with guidance from research staff (E). (4) Incentive payments (E) → less alcohol consumption per day	Not studied	5
**Kuerbis et al. (2015); USA** [41]	N = 86; >50+ years Mean age 64.7 (8.4)	Quantitative randomized controlled trial	*Intervention*: Brief mailed intervention with personalized mailed feedback outlining their specific risks associated with alcohol and educational booklets *Control*: No intervention	*Intervention*: (1) A personalized feedback report (E) and (2) two alcohol education booklets (E) → less at-risk drinking (O)	Not studied	4
**Lockwood et al. (2020); UK** [92]	N = 3057 (questionnaires) N = 14 (interviews) Age between 45–65 years	Mixed methods questionnaires and interviews	*Aim:* evaluate the impact of a “gain-framed”, multimedia campaign to encourage heavier drinking men aged 45–64 years to drink less.	*Intervention:* more aware of how much they routinely drink, and to make healthier choices. (1) Providing information about health consequences (E); providing information about emotional consequences (E); encouraging self-monitoring of behavior (E); encouraging self-monitoring of outcomes of behavior (E) and encouraging behavioral experiments (E).	Appreciated the friendly, non-threatening tone and that the message was straightforward (M), meaningful, achievable (M), and was gainframed—i.e., emphasised the benefits of drinking less rather than the harms of drinking too much (M) → reduction of alcohol consumption (O)	4
**Moody et al. (2018); USA** [93]	N = 36; 18–65 years Intervention: mean age 38.89 (11.58) Control: mean age 40.24 (12.91)	Quantitative non-randomized	*Intervention*: Two-week implementation intention interventions that linked high-risk situations with alternative responses *Control*: Two-week implementation intention interventions for selected situations and responses but did not link these together	*Intervention*: (1) Cut back on drinking over the following two weeks (E) (2) and fill in an “if–then” worksheet format. Response (linked high-risk situations with alternative responses) (E) → with a significant reduction in alcohol consumption when drinking was reported (O) → more abstinent days (O) *Control*: To try to cut back on drinking over the following two weeks (E) and (3) asked to select situations and responses but did not link these together (E) → more abstinent days (O)	Not studied	3
**Nygaard (2001); DK** [94]	N = 13; 35–45 years	Qualitative interviews	*Intervention*: The participants were asked to abstain from drinking alcohol for 6 weeks, during which period they were to maintain their “normal” social behavior and obligated to keep a diary of their experiences with abstinence	*Intervention*: (1) Abstain from drinking alcohol for 6 weeks (E), during which period participants were to maintain their “normal” social behavior (E) → the participants reporting the largest decrease in consumption were the persons reporting the highest initial consumption level (O)	*Intervention*: (1) Abstain from drinking alcohol for 6 weeks (E), during which period the participants were to maintain their “normal” social behavior (E), producing increased awareness of the role of alcohol in their lives (M). (2) Participants expressed more insights into their expectations of social gatherings and how to fulfil them (M) → the participants reporting the largest decrease in consumption were the persons reporting the highest initial consumption level (O). (3) More participants reported that they now made conscious decisions about their alcohol consumption prior to participating in a social gathering and that they would feel more comfortable complying with those decisions (M) → some started to drink at a slower pace, and others started bringing their own water bottles (O)	3
**Van Lettow et al. (2015); NL** [95]	N = 2634; Age not specified Mean age 37.03 (15.19)	Quantitative randomized controlled trial	*Intervention 1*: Drinktest (online personalized feedback intervention) plus prototype alteration (feedback regarding prototype alteration tailored to gender, drinking behavior (also including normative feedback), intentions, and prototypical self-characterization) *Intervention 2*: Drinktest (online personalized feedback intervention) plus cue reminder *Intervention 3*: Drinktest (online personalized feedback intervention) plus prototype alteration and cue reminder *Control*: Original Drinktest (1) received feedback tailored to demographic background (gender drinking behavior (also including normative feedback), intentions, and prototypical self-characterization), alcohol consumption and intentions to reduce drinking. These messages reflected on personal drinking levels in comparison with the Dutch norm and peers’ drinking behavior	*Intervention 1*: (1) Received feedback tailored to gender, drinking behavior (also including normative feedback) (E), intentions and prototypical self-characterization; (2) the prototype message reflected on characteristics that the participants evaluated as personally desirable or undesirable by evaluating themselves on 11 characteristics (E); (3) participants were encouraged to reduce their drinking to achieve their desired characteristics and, in turn, to be positively valued by peers (E); (4) then, participants were guided in their goal setting by selecting an action plan to achieve the desired characteristics (E) → reduction of alcohol consumption (O) *Intervention 2*: (1) Received feedback tailored to demographic background (gender), alcohol consumption and intentions to reduce drinking. These messages reflected on personal drinking levels in comparison with the Dutch norm and peers’ drinking behavior (E). Participants were guided in their goal setting by selecting an action plan to achieve the desired characteristics (E). (2) Feedback was provided that reflected on their action plans, explaining that a cue reminder may help them to remember their plans (E) (if made) and they received a free silicone bracelet by mail. If participants did not want to receive the bracelet, they were encouraged to select a piece of their own jewellery or another object of frequent use (E) → reduction of alcohol consumption (O) *Intervention 3*: (1) Drinktest plus prototype alteration, cue reminder and feedback tailored to gender, drinking behavior (also including normative feedback) (E), intentions and prototypical self-characterization. (2) The prototype message reflected on characteristics that the participants evaluated as personally desirable or undesirable by evaluating themselves on 11 characteristics (E); (3) participants were encouraged to reduce their drinking to achieve their desired characteristics and, in turn, to be positively valued by their peers (E). (4) Participants were guided in their goal setting by selecting an action plan to achieve the desired characteristics (E). (5) Feedback was provided that reflected on their action plans, explaining that a cue reminder may help them to remember their plans (E) (if made) and they received a free silicone bracelet by mail. If participants did not want to receive the bracelet, they were encouraged to select a piece of their own jewellery or another object of frequent use (E) → reduction of alcohol consumption (O) *Control group*: Original Drinktest: (1) received feedback tailored to demographic background (gender), alcohol consumption and intentions to reduce drinking. These messages reflected on personal drinking levels in comparison with the Dutch norm and peers’ drinking behavior → reduction in alcohol consumption (O)	Not studied	3
**Tait et al. (2019); AUS** [96]	N = 793; >18+ years Mean age 40.1 (10.0)	Quantitative randomized controlled trial	*Intervention 1*: Daybreak is a self-guided programme, accessible via mobile app and desktop with weekly check-ins and peer support. The Daybreak programme enables participants to connect with other users of the programme through a blog function *Intervention 2*: Daybreak + coaching: Daybreak and access to an online health coach between 7:00 and 19:00 on weekdays	*Intervention 1*: (1) Weekly check-ins: the programme includes self-reported questionnaires to encourage participants to undertake self-reflection to explore their intrinsic motivators for change (E) (2) Peer support: the programme enables participants to connect with other users of the programme through a blog function (E) → reduction of alcohol use (O)*Intervention 2*: No effective elements	Not studied	3
**Zill et al. (2019); DE** [97]	N = 608; >18+ years Intervention: mean age 40.4 (11.2) Control: mean age 40.7 (12.1)	Quantitative randomized controlled trial	*Intervention*: Vorvida: a German Internet intervention based on cognitive behavioral therapy (CBT) methods, which automatically tailors content to match individual user characteristics	*Intervention*: (1) Internet intervention based (E) on (2) cognitive behavioral therapy (E) methods, which (3) automatically tailors content to match individual user characteristics (E) → less alcohol consumption (O) → less binge drinking (O)	Not studied	4
**F. Context: No Therapist—Not In-Person—Group Component**						
**Author; Country**	**Participants; Age** **Mean (SD)**	**Method**	**Intervention or Aim**	**Intervention Elements (E) ^2^ and Outcome (O) ^4^**	**Mechanisms (M) ^3^ and Outcome (O) ^4^**	**Study Quality (MMAT)**
**Black et al. (2020); AUS** [98]	N = 24 Age not specified Mean age: 42.42 (8.69)	Qualitative interviews	*Aim:* to inform recruitment and retention strategies by exploring users’ motivations and experiences in using a novel, Internet intervention, the Hello Sunday Morning (HSM) program.	*Intervention*: (1) Publicly set a personal goal to stop drinking or reduce consumption for a set period of time (E); (2) record their reflections and progress on blogs and social networks (E) → reduction of alcohol consumption (O)	Support and normalization: participants gained social support from other consumption (M), and their problems with alcohol and desire to seek help were normalized (M); (2b) goal setting and self-monitoring: setting goals (M) and monitoring progress provided participants with motivation and self-accountability (M) → reduction of alcohol consumption (O)	5
**Haug et al. (2020); USA** [99]	N = 57 Age 21–30, 31–40, 41–50, 51–60, 61 or older	Quantitative descriptive	*Intervention:* Self-guided alcohol Internet intervention that provides access to several different online social networks and is based on principles of harm reduction, cognitive-behavioral therapy (CBT), and relapse prevention	*Intervention:* Online mutual help program to change their alcohol drinking(E) cyber community (E), social networking (E), and self-help tools (E) → reduction of alcohol consumption (O)	consumption of more than one online activity (e.g., Facebook group plus online chat) (M) was associated with greater reductions in self-reported alcohol consumption (O)	4
**Kirkman et al. (2018); AUS** [44]	N = 1917; Age not specified Mean age 46 (11.71).	Quantitative non-randomized	*Intervention Hello Sunday Morning* (*HSM*): An Australian social media health promotion “movement” that asks participants to set a personal goal publicly to stop drinking or reduce their consumption, for a set period of time, and to record their reflections and progress on blogs and social networks	*Intervention*: (1) Publicly set a personal goal to stop drinking or reduce consumption for a set period of time (E); (2) record their reflections and progress on blogs and social networks (E) → reduction of alcohol use (O)	Not studied	1

^1^ Context (C): the way in which the intervention is offered to the target group. ^2^ Intervention elements (E): the elements from an intervention that contributed to the desired outcome. ^3^ Mechanisms (M): the responses of people regarding the intervention elements. ^4^ Outcome (O): reducing or abstaining from alcohol consumption. * The study is mentioned twice in the table because of the two different interventions. Walitzer and Dermen (2004); USA [61], Kiluk et al. (2016); USA [53].

### 3.2. Themes

We were interested in *how* (which elements of interventions), *in which context* and *why* (which mechanisms) interventions prevent or reduce (problematic) alcohol consumption among older adults. The results were first categorized according to their mode of delivery (i.e., the context): (1) practitioner or no practitioner involvement; (2) in-person or not; and (3) individual treatment, group treatment or treatment with relatives’ involvement. Consequently, six different modes of delivery were found: (A) practitioner—in-person—individual; (B) practitioner—not in-person—individual; (C) practitioner—in-person—relatives; (D) practitioner—in-person—group component; (E) no practitioner—not in-person—individual; and (F) no practitioner—not in-person—group component. Then, for every mode of delivery, one or more findings were provided about how (which elements of interventions) and, when found, why (by which mechanism) these elements contributed to the prevention and reduction of (problematic) alcohol consumption for (older) adults. Table 4 provides a summary of the studies’ characteristics. Table 5 provides a summary of the results.

**A. Practitioner—in-person—individual**

*Paying attention to drinking behavior*

From the treatments that were delivered by a practitioner, in-person and individually, four effective elements were present: (1) motivational exercises [53,56,60]; (2) pointing out the health disadvantages of drinking behavior [51,52,79]; (3) helping to develop networks [54,57,58,60]; or a combination of these approaches [46]. Paying attention to drinking behavior yields results. Interventions make people think and act differently about alcohol consumption [57] and seek help from family and friends [57,58]. In many studies, the drinking behavior of the control group also changes, although they receive a much smaller intervention [45,53] or no intervention at all [79].

*The relationship between the patient and the therapist*

The relationship between the patient and the practitioner is of great importance for a successful outcome of the treatment [42,47,49,50,59]. More treatments can improve the relationship between patient and therapist [59]. If the practitioner shows certain behavior [42,47,50], such as reflective listening to the patient, the relationship also improves. There are also indications [47,50] that, if the patient and the practitioner collaborate in the identification of additional sessions judged best to meet the patient’s clinical needs, the relationship improves and alcohol consumption is reduced.

**B. Practitioner—not in-person—individual**

*Personal contact and feedback*

Of the treatments that were delivered by a practitioner, via telephone or online and individually, five effective elements were present. If a counselling session is given over the phone by a practitioner and a (1) workbook is sent out afterwards on how to reduce alcohol consumption [39,61] or if (2) personalized feedback is given before or after the telephone sessions [39,62,64], then drinking behavior is reduced, also among older adults. (3) If an in-person session is followed by a phone call [65], this also helps to reduce drinking behavior.

*Online communication and feedback*

If treatment is given via online communication by means of (4) assignments or modules undertaken by the participant about his or her drinking behavior followed by a chat session with the practitioner about the assignments [63] or is (5) followed by feedback from the practitioner [66], then the drinking behavior is reduced. For none of these elements were the reasons why they were effective and which mechanisms they triggered found.

**C. Practitioner—in-person—relatives**

Regarding the treatments that were delivered by a practitioner, in person and included the involvement of relatives, two effective elements were found.

*The status of the relationship*

The (1) status of the relationship with (marriage) partners/family members influences the outcome of the intervention [61,68,70,71,72,73]. By influencing this status, the treatment can also lead to a successful outcome [69,71].

*Teaching the partner to deal with drinking behavior*

The partner can be (2) taught to deal with the drinking behavior of the partner through therapy [61,69,70,72] or through (video) information [68], which can lead to lead to alcohol reduction of the drinking partner. If the non-drinking partner is taught to deal with the behavior of the drinking partner, this can lead to more understanding and support from the non-drinking partner for the drinking partner [69]. The drinking partner is then better advised not to use alcohol or to moderate alcohol consumption.

**D. Practitioner—in-person—group component**

Of the treatments that were delivered by a practitioner, in-person and in a group setting or in a group setting at work, two effective elements were present.

*Motivating to change lifestyle*

Brief group interventions focusing on (1) motivating participants to change their lifestyles regarding personal relationships, nutrition and exercise [79] and coping with desires for alcohol [65,74] lead to alcohol reduction.

*Motivating to change lifestyle delivered in a workplace setting*

If an intervention is given in a work setting in which (1) alcohol use and its consequences are discussed [76,77,78] and/or in which a (2) training element is offered that intends to change behavior and reduce alcohol use [76,77,78] and/or (3) personal advice is given on alcohol use [76], this leads to lower (risky) alcohol use. For none of these elements were the reasons why they were effective given.

**E. No practitioner—not in-person—individual**

In relation to treatments that were not delivered by a practitioner, were not in-person and were individual, five effective elements were present.

*Web-based interventions*

Web-based interventions that give (1) personal feedback [43,64,81,85,86,89,90,95,97] and of which the respondents’ result is also (2) compared with the results of people who are in the same phase [43,81] or have the same age group, gender or country of origin [85,89,95] or is compared with the previous data of the participant [81] ensure lower alcohol consumption. Web-based interventions based on cognitive behavioral therapy (CBT) that (3) gradually teach the participant skills for refusing drinks, dealing with cravings, etc., result in lower alcohol consumption [53]. Web-based interviews for older adults that also contain elements of personalized feedback and complement this with information on each person’s own specific risks of alcohol consumption as well as information on the effects of alcohol on health, medication use and functional status and recommendations for safe drinking [40,41] lead to lower alcohol consumption.

*Telephone based interventions*

When a (mobile) phone intervention consist of a (1) self-guided program or modules or steps in which coping strategies and control functions for many alcohol-related issues are taught [83,87,88,89,96], this could lead to less alcohol consumption and less binge drinking. Mobile phone interventions provide insight into how much someone drinks and leads to realization of their own drinking behavior [87,88]. The provision of (2) self-help material on the consequences of alcohol use and motivating behavioral change in relation to alcohol use [84] during a telephone-based intervention leads to less alcohol use.

**F. No practitioner—not in-person—group component**

In the treatments that were not delivered by a practitioner, not in-person and in an online group setting, two effective elements were present: Intervention of abstinent people (with or without problematic drinking behavior) (1) from drinking alcohol for a certain period or to drink less [44,98,99] and (2) to share this experience with peers [44,98] makes people aware of their alcohol consumption and reduces alcohol consumption.

## 4. Discussion

We were interested in *how* (which elements of interventions), *in which context* and *why* (which mechanisms) interventions prevent or reduce (problematic) alcohol consumption among older adults. We found information on the functioning of alcohol interventions for the general population (which often were designed for an 18+ population and therefore also included older adults). Three effective elements of interventions were identified in several types of contexts for the general population. Two of these three effective elements were also found in the interventions especially designed for older adults.

The first element that was mentioned in almost all the contexts was the *provision of information on several alcohol-related issues:* the health disadvantages of drinking behavior [40,41,51,52,79]; coping strategies and control measures for many alcohol-related issues [39,63,76,77,78,83,84,87,88,89,96]; and changing participants’ lifestyles regarding personal relationships, nutrition and exercise [82].

The second effective element was *being in contact with others and communicating with them about* (*alcohol*) *problems.* Sometimes practitioners help participants to develop (new) social networks [46,57,58,60]. Sometimes the family members or partners of the participants are taught to understand the drinking habits of their loved ones and how to support them in drinking less or abstaining from drinking [70,72]. Contact with peers and colleagues is also an important factor. Participants have to share their experience of abstinence for a period with their peers [44,94] or discuss with their colleagues, in a work setting, alcohol use and its consequences [76,77,78]. The importance of the role of contact with others or *social networks* on alcohol consumption has been acknowledged previously [100,101]. The A study by Robinson et al. [102] showed strong negative associations between empathic processing (the thoughts or feelings of others and responding accordingly) and social support and both the consequences of drinking and the percentage of drinking days.

Providing participants with *personalized feedback* about their drinking behavior is the third commonly found effective element across the context settings. This element leads to results in interventions that are given by a practitioner in-person [67,76] or by a practitioner via telephone [39,63,65] but also when the feedback is provided through computer-generated communication [40,41,43,64,81,85,86,89,95,97]. The effect of personalized feedback on alcohol consumption was described as important in an earlier review of online alcohol interventions [103]. The study by Riper et al. [104] showed that single-session, individually personalized feedback without professional guidance can be effective in reducing risky alcohol consumption in young and adult problem drinkers.

The element of the *provision of information* on several-alcohol related issues was also found among one of the three interventions especially designed for older adults [39]. In addition, the element of *providing personalized feedback* was found in two of the three interventions for older adults [40,41]. The element of *contact with others* was not found in the three interventions especially designed for older adults. This is striking because contact with others is especially important for older adults since loneliness is a problem for that age group [105] and there is a relationship between the use of alcohol and loneliness [12,106,107].

We only found three studies on the prevention or reduction of alcohol consumption that were specifically designed for older adults. The reason for this low number of studies could be that the results of the aging of the population (people in general are becoming older and the absolute number of older adults is rising) have only become clear in the last few years and will increase in the years to come. The importance of research into the reduction and prevention of alcohol use in older adults has only recently become more apparent.

### 4.1. Limitations

We did not include grey literature in our review because our aim was to give an overview of the scientific peer-reviewed literature on interventions for older adults to reduce or prevent (problematic) alcohol use first. If we had included grey literature, we might have found more interventions designed specifically for older adults. Although we included many randomized controlled trials, we could not perform a meta-analysis because of the heterogeneity among the interventions, the study populations and the results. We chose to limit the operationalization of the context to the mode of delivery to make it easier to compare the contexts of the studies. For many studies, other information about the context was scarce or incomplete. If this information had been provided, a better comparison of contexts would have been possible. We only included Western high-income countries since problematic drinking behavior is highest among the population in these countries. Non-western countries were excluded because drinking culture, and thus also offered interventions to older adults, differs from western countries. This may limit the generalization of this study to other countries. Results can be generalized to the general (older) population, but not to specific groups (e.g., pregnant people, veterans) since drinking culture is different among these sub-groups. Future research might investigate other vulnerable subgroups. Another limitation is that not in all articles was the ‘why’ mechanism addressed, indicating that a complete overview of why some interventions were effective is lacking in current research reports. Future research about why interventions were effective and especially why interventions are effective for older adults is necessary. Despite the limitations, this study provides a broad overview of which elements of interventions are effective in preventing or reducing alcohol use as well as indicating why these elements are effective.

#### 4.1.1. Practical Implications

This literature review identified three major effective elements of interventions: (1) providing information on the consequences of alcohol consumption; (2) being in contact with others and communicating with them about (alcohol) problems; and (3) personalized feedback about drinking behavior. Two of these elements, information provision and personalized feedback, are related to creating awareness. This is also a common answer to why an intervention works. People became aware of their alcohol consumption and what it means for their bodies. For developers of new interventions concerning the reduction or prevention of alcohol consumption of (older) adults, but also for policy makers, it could be a good start to look at what creates awareness regarding alcohol consumption for that specific target group. The third effective element, contact with others and communicating about (alcohol) problems, is also an element that is important for developers of interventions and policy makers. People explain that sharing their experiences of (reducing) alcohol consumption helps them. In doing so, it is important that friends and family are supportive of the choice of the person to reduce or stop drinking and respond empathically about this choice. This could be difficult for some friends or family members as drinkers tend to seek each other out and then influence each other’s use [100]. Developers of interventions and policy makers could therefore facilitate the process of helping (older) adults to develop contacts with people that are supportive of their choice to reduce or prevent their alcohol consumption.

#### 4.1.2. Scientific Recommendations

We only found three studies on the prevention or reduction of alcohol consumption that were specifically designed for older adults. In order to provide adequate interventions to help reduce or prevent alcohol consumption for older adults, more research is necessary on what creates awareness regarding alcohol consumption for this target group. Moreover, research on how to help older adults develop contacts with people that are supportive of their choice to reduce or prevent their alcohol consumption is necessary, because these contacts are helpful in reducing or preventing alcohol consumption.

## 5. Conclusions

This study provides answers to the questions of how (which elements of interventions), in which context and why (by which mechanisms), interventions prevent or reduce (problematic) alcohol consumption among older adults. Most of the studies were not especially designed for older adults but also included older adults. The findings of this study highlight three major effective elements of interventions: (1) providing information on the consequences of alcohol consumption; (2) being in contact with others and communicating with them about (alcohol) problems; and (3) personalized feedback about drinking behavior. Two of these elements were also used in the interventions especially designed for older adults. In order to provide adequate interventions to help reduce or prevent alcohol consumption for older adults, more research is necessary on what creates awareness regarding alcohol consumption for this target group.

## Figures and Tables

**Table 1:** Databases.

Database	Limits
PsycINFO	Peer-reviewed journals, age group: adulthood 18+, English, Dutch, years 2000–2022
Web of Science (WOS)	Articles, publication years 2000–2022, English, Dutch ^1^
PubMed	Adult: 19+ years, ^2^ publication years 2000–2022, English, Dutch
CINAHL	Peer-reviewed, publication years 2000–2022, English, Dutch ^1^

^1^ No limits for age were provided. Therefore, the word group “Not child” was used. ^2^ The only age limit that was provided was 19+.

**Table 2:** Groups of key words.

**Group 1 Alcohol**
alcohol or “alcohol consumption”
**Group 2 Older adults**
elder or elderly or senior or old or pension or retire or retirement or “later life” or geriatric or geriatrics or “older adults” or ageing or aging or gerontology or aged
**Group 3 Reduction**
intervention or treatment or reduction
**Group 4 Prevention**
prevent or prevention or preventing
**Group 5 Not child**
NOT child or “young adult” or teenage or adolescent

**Table 3:** Search strings.

Search Questions	Groups of Keywords
What are the effective elements of interventions for the **general population** with regard to **reducing** (problematic) alcohol consumption?	1 (*title*) and 3 (*title*) not 5 (only for WOS (*topic*) and CINAHL (*title*))
What are the effective elements of interventions for **older adults (55+)** with regard to **reducing** (problematic) alcohol consumption?	1 (*title*) and 2 (*title*) and 3 (PsycINFO (*abstract*), WOS (*topic*), PubMed (*title/abstract*), CINAHL (*abstract*))
What are the effective elements of interventions for the **general population** with regard to the **prevention** of (problematic) alcohol consumption?	1 (*title*) and 4 (*title*) not 5 (only for WOS (*topic*) and CINAHL (*title*))
What are the effective elements of interventions for **older adults (55+)** with regard to the **prevention** of (problematic) alcohol consumption?	1 (*title*) and 2 (*title*) and 4 (PsycINFO (*abstract*), WOS (*topic*), PubMed (*title/abstract*), CINAHL (*abstract*))

**Table 5:** Summary of the results.

Context	Element of Intervention (How)	Mechanism (Why)	Outcome
**A. Practitioner—in-person—individual**	*Paying attention to drinking behavior* (1) motivational exercises to change behavior (2) pointing out the health disadvantages of drinking behavior (3) helping to develop networks	Interventions make people think and act differently about alcohol consumption and seek help from family and friends	Less or no alcohol consumption
	*The relationship between the patient and practitioner* (1) Empathic behavior of therapist	Patient and practitioner collaborate in the identification of additional sessions, judged best to meet the patient’s clinical needs and the relationship between the patient and the therapist improves	Less or no alcohol consumption
**B. Practitioner—not in-person—individual**	*Personal contact and feedback* (1) workbook, (2) personalized feedback (3) follow-up telephone calls	-	Less or no alcohol consumption
	*Online communication and feedback* (1) assignments or modules (2) follow-up chat session		
**C. Practitioner—in-person—relatives**	*The status of the relationship*	-	Less or no alcohol consumption
	*Teaching the partner to deal with drinking behavior*	When the non-drinking partner is taught to deal with the behavior of the drinking partner, this can lead to more understanding and support from the non-drinking partner for the drinking partner.	
**D. Practitioner—in-person—group component**	*Motivating to change lifestyle* (1) regarding personal relationships, nutrition and exercise (2) and coping with desires for alcohol	-	Less or no alcohol consumption
	*Motivating to change lifestyle delivered in a workplace setting* (1) discussion of alcohol use and its consequences (2) training element to change behavior and reduce alcohol use (3) personal advice is given on alcohol use		
**E. No practitioner—not in-person—individual.**	*Web based interventions* (1) personal feedback (2) comparing own results with others (same phase, age group, gender or country) (3) compared with the previous data of the participant. (4) cognitive behavioral therapy (CBT)	-	Less or no alcohol consumption
	*Telephone based interventions* (1) self-guided programme or modules or steps on coping strategies and control functions (2) self-help material on the consequences of alcohol use and motivating behavioral change	Mobile phone interventions could provide insight into how much someone drinks through the information provided and this leads to realization of their own drinking behavior	
**F. No practitioner—not in-person—group component**	*Intervention to abstinent people* (1) Not drinking alcohol for a certain period or to drink less (2) share this experience with peers	Intervention to abstinent people (with or without problematic drinking behavior (1) from drinking alcohol for a certain period or to drink less and (2) to share this experience with peers makes people aware of their alcohol consumption and reduces alcohol consumption.	Less or no alcohol consumption

## Data Availability

Not applicable.
